# Foreign

**Published:** 1840-09-26

**Authors:** 


### FOREIGN.

*Instrument for treatment of Myopia.* By Aug. Franz, M. D.—In reading the *Allgemeine Zeitung* of May the 7th, I first became acquainted with a new method of treating myopia by means of an apparatus invented by Dr. Berthold, professor at the University of Göttingen. To receive more explicit information on a subject of so much importance as this discovery appeared to me, I placed myself in direct correspondence with Professor Berthold, and to his kindness I am indebted for the means of laying this report before the profession, as he not only sent me several communications by letter, but also the paper read by him before the Royal Society of Sciences at Göttingen, accompanied with a drawing of the apparatus. On the leading points I give short extracts from his paper and from his letters.

"In any attempt to cure myopia," says Professor Berthold, "our attention must first be directed to the *mutationes oculi internæ*, or the faculty inherent in the eye, of accommodating itself to the various distance of objects from the eye. Of the existence of this faculty in the eye there can be no doubt, but in what this power of adjustment consists is a question upon which a great diversity of opinion yet prevails. At all events it is subject to the will. According to the calculations of Olbers, the proportion of this power of adjustment in the human eye is such, that if the distance of the crystalline lens from the retina could be altered to one line, we should be enabled to see objects with equal distinctness, from a distance of four inches to the utmost extent of human vision. The same effect would result from a displacement of the lens to a distance of one-half of a line only, provided the radius of the cornea could be altered to about two-fifths of a line.

" The chief indication in the cure of myopia is to accommodate the above-mentioned vital power of adjustment to the physical power of refraction in the transparent parts of the eye; and to the attainment of this end, generally speaking, no obstacle exists. To regulate this power of adjustment so as to effect a permanent cure, or at least a diminution of myopia, we must proceed upon the principle that, like every other voluntary motion of the body, it can be increased by continued and proper exercise, and by, at the same time, carefully obviating whatever might interfere with the advantages gained by it. With a view to attain this object, an apparatus, called the *myopodiorthoticon*,* has been invented, which serves for the purposes of reading and writing; these being the means best adapted for the cure of myopia as intended by this instrument.

" This apparatus consists of a desk to be placed upon a table. The desk is articulated anteriorly with a board or pedestal of equal size by means of hinges, with a view of regulating and adjusting it to a proper position, in which it may be retained by a support. From the posterior part of the desk two screws rise vertically, one at either side. These screws pass through a cross-bar, which may be moved upwards or downwards by means of a mother-screw situated below it, and retained in this position by a second mother screw, situated above it. Through the centre of the cross-bar a graduated rest passes, in a horizontal direction, for the support of the head, which rest, being moveable, is held in position by a screw. By the motion of the desk upon its pedestal the cross-bar on the lateral screw, and the rest in the cross-bar, the apparatus may be so regulated, that a book to be read may be brought in the position best adapted to the situation of the eyes, and the power of sight. Parallel to one of the lateral screws of the desk a scale passes upwards through the cross-bar. This scale is graduated like the above-mentioned rest, that the gradual diminution of the near-sightedness may be accurately ascertained, but more especially that the apparatus may be adjusted to the difference in the print of the books read."

The rules to be observed in the employment of this instrument are, according to Professor B., chiefly these :—

" 1. The apparatus must be so regulated that the person using it can read large print with ease whilst leaning with the upper part of the root of the nose against the free extremity of the rest previously brought in such a position as to place the eyes as accurately as possible opposite the centre of the book upon the desk. Care must be taken, at the same time, that the one-half of the book containing a smaller number of leaves have its deficiency compensated by suitable support, so that both halves may always form a horizontal plane.

" 2. In moving the cross-bar upwards, by turning the mother-screws on the lateral screws, the distance from the free extremity of the rest to the book, or the distance of distinct vision, is to be increased every second, third, or fourth day, according to the scale, about one-half or a whole line, but never to a point where the print can no longer be read with facility, and where, therefore, reading would become an exertion to the eyes. A rapid increase of distance must be especially avoided, as the power of adjustment in the eye increases but slowly, and would not be able to follow ; and, moreover, too great an exertion of this power can even become hurtful to the organ.

" 3. For reading with the apparatus, a book must be chosen of a clear and large print, and, if possible, with the same sized type throughout, not interspersed with italics, &c. The same print must be read as long as possible ; a work containing a large number of volumes is, therefore, preferable. Should a change of book, or of the type become necessary, the distance of the rest from the book must be regulated again to that point where the print can be read with ease, and the increase of distance gradually conducted in the manner above mentioned. For writing, the distance of the rest from the paper placed upon the desk may be somewhat more ample than for reading, if the patient observes that general rule for myopic persons, viz., to write a large hand."

4. " During the progress of the treatment, all occupations requiring close or irregular approximation of the eyes to the object, such as sewing, knitting, embroidering, drawing, &c., must be avoided. At times, when the patient is not using the apparatus, he should practise looking at distant objects only, take frequent exercise in the open air, and view distant trees, green fields, and hills. A proper diet should be observed, and all heating food and liquors abstained from; a cooling aperient is to be taken now and then, and habitual congestion of the head or eyes suitably counteracted."

Professor Berthold states in one of his letters to me, that in Göttingen, as well as in several other parts of Germany, this instrument is already much in use for the treatment of myopia, and that its effect has surpassed his expectation, even in the most severe cases. Of the numerous cases under his treatment he relates one of a student, who was very anxious

* Derived from *μυωψ*, a near sighted person, and *διορθωτικόν*, something apt or fitted to improve.

to be cured of his myopia, or to have his sight at least improved. This young man, aged twenty-six, enjoyed very good sight from his birth, but during his education at school this had gradually diminished until a confirmed myopia established itself. When the patient placed himself under the Professor's care, the degree of near sightedness was such, that he could only read a common print at a distance of four inches ; he suffered at the same time from a congested state of the vessels in the conjunctiva and eyelids, the margins of which were rather swollen, and easily reddened by the stimulus of light and air. A few leeches were applied in the neighbourhood of the eyes, and occasionally repeated. Sulphur, cremor tartari, and pulvis rhei, ordered to be taken internally, so as to ensure two or three loose motions daily, and a strict diet recommended. After the continuation of this treatment for a short time, the myopodiorthoticon was brought into use; by its use the distance of vision became gradually increased in the manner above described, so that the patient now, after the lapse of scarcely four months, can read with facility and distinctness, at a distance of eleven inches and one-third, a print which formerly he could only read at four inches distance. He also sees distant objects in the open air far better than before. The use of the apparatus is still continued, and he is not content with the distance of eleven inches and one-third, but purposes extending his sight to sixteen or eighteen inches. When the patient commenced the use of the apparatus he had made rapid progress in increasing the distance of vision, by which he had certainly gained great practice in the power of adjustment of the eyes, but this organ having been over-exerted became easily fatigued, so that it was considered necessary to begin the use of the instrument again from the commencement. Prof. B. lays it down as a rule never to be deviated from, that a slow and steady progressive increasing of the distance of vision, especially in the beginning of the treatment, is the chief condition upon which a safe and perfect removal of myopia by this apparatus depends. He farther remarks, that in early life a more rapid cure may be expected than in mature age; and that the time of duration of the treatment with the apparatus is exactly proportioned to the degree of myopia.

I have been informed by several of my friends in Germany, that the Royal Society of Sciences of Göttingen has highly commended the ingenious construction of the myopodiorthoticon, and expressed their approbation of this new method of treating nearsightedness ; and that much attention and interest have been excited, not only at Göttingen, but also in several other towns of Germany, by this invention. If we attentively consider the principles upon which the construction of this instrument rests, we shall find that they are theoretically perfectly correct, and therefore, in my opinion, its practical worth can hardly be doubted, the less as its inventor has already succeeded in curing a number of cases of myopia. There appears even every reason to believe that a judicious use of this instrument will prove effective not only in myopia, but also in cases where presbyopia exists in young persons, or where one eye is near and the other farsighted—an ophthalmic defect which is not very rare.

As this instrument is very simple in its construction, any writing-desk used in colleges, offices, &c., may be easily furnished with its more essential parts, and thus students, clerks, &c., afflicted with nearsightedness, which, in these persons, is always brought on by their daily occupation of reading and writing, may make the cause of their affection the means of cure. Proper attention must of course be paid to the medical and general rules laid down for this method of treating myopia. This instrument may, further, be a means of preventing myopia, when used at an early period of life, especially by those children in whose eyes an hereditary predisposition for myopia may exist, which generally shows itself after the commencement of instruction. For this purpose the apparatus might be introduced into schools and other public institutions for education on a large scale, viz., by converting a whole school-table into a common myopodiorthoticon, which is regulated according to the distance of vision of each individual, chiefly by means of a somewhat different rest for the support of the head. The introduction into public institutions of the myopodiorthoticon thus contrived would moreover prove advantageous in several other respects, inasmuch as the use of this apparatus would oblige the children to sit in an upright position, and to hold their heads erect, and thus prevent curvatures of the spine, complaints of the chest, congestions of the head, and other diseases, which are apt to appear in children of a weakly constitution, after an habitually improper position of the upper part of the body during reading and writing.

I have now a myopodiorthoticon in my possession, which I had made according to Professor Berthold's drawing, with the exception only, that instead of one graduated scale, I ordered two to be made, one at each side of the desk, in order to be enabled to regulate the cross-bar with greater precision. The pedestal I have also altered, and it is now so contrived that, while the whole instrument remains upon the table, the desk may be raised to a height suitable for a person whilst sitting or standing. With this instrument I have written and read myself during the last four weeks, merely to ascertain whether its use is attended with inconvenience or restraint; but I find that, on the contrary, when once habituated to it, much comfort and support are derived from its employment.

As perhaps mine is the only instrument of this description in this country, I shall feel great pleasure in affording to those who may be interested in this method of treating nearsightedness, the opportunity of examining the mechanical contrivance upon which this method is based.—*London Medical Gazette.*

*New Method for the Radical Cure of Varix, and especially of Varicocele.* By M. Ricord.—After pointing out the errors of believing that varicocele affects only persons of twenty or thirty years old, and imagining it to be a common consequence of gonorrhœa or epididymitis, whereas in fact it is more generally a predisposing cause of the latter disease, and instead of being produced by it, is more often cured by it; M. Ricord proceeds to describe his mode of operation.

"The hair must be shaved from the genital organs on the side to be operated on, and the veins must be dilated by making the patient walk about a little, or by enveloping the scrotum for a few hours in hot poultices, or by fomentations. This being done, (though if the swelling is at all times considerable these precautions are unnecessary,) the vas deferens must be separated from the mass of veins, and the latter being taken up with a fold of the scrotum, a flat, lance-shaped needle, armed with a double-looped thread, must be passed beneath them. When the needle has been passed completely through the skin from one side to the other, the veins are to be let go, the skin alone being now held up, and then a second needle similarly armed must be passed through over the veins, entering at the same hole by which the first needle was thrust out, and passing out at the same hole by which the first entered. The bundle of veins is thus fixed between two double threads, of which one passes over and the other beneath it. The ends of each double thread on each side are then to be passed into the loop of the other, and now by drawing these ends in opposite directions the vessels are tied beneath the skin. By this kind of ligatures the vessels may either be suddenly constricted or be tied gradually in a manner something like that adopted by M. Breschet, or most conveniently by a properly adopted *serre-nœud* after the fashion of a tourniquet.

"It is usually from the tenth to the twentieth day that the vessels are divided by this means, and their division may be easily recognised by the freedom with which the ligatures may be drawn from one side to the other without being, as they were before, retained by the parts which they inclosed. It sometimes happens that at the instant of the first constriction the patient suddenly feels rather an acute pain in the course of the spermatic cord; it is usually less severe than in the other operations for the same purpose; and though it often recurs at the successive constrictions, yet it has never been long continued, nor given rise to any accident. It is sufficient to keep the scrotum raised, to employ some anodyne frictions on the inguinal canal or the lumbar region, or to apply some emollient poultices, to effect its removal. Sometimes a slight œdema of the scrotum supervenes, and I have twice observed rather a considerable serous effusion in the tunica vaginalis. In one patient also, who went out of the hospital, and a few days after the operation exposed himself to great fatigue, a slight abscess formed in the cellular tissue; but with these exceptions there has been no important accident.

"It must be clearly understood that if the patient is strong and plethoric he is to be bled from the arm directly after the operation; that the horizontal position must be maintained till the vessels are cut through; and that the bowels must be carefully kept open.

"Twelve patients have now been operated on in this manner at the Venereal Hospital, and in all the most complete and satisfactory result has been obtained. The three last of them were presented at the Academy of Medicine; two completely cured, and the third, who was operated on only two days previously, still wearing the ligature and the *serre-nœud.*

"I have employed the same method for varices of the legs. I have already operated on nine patients, some having simple varicose swellings, and others varicose ulcers. In some a single ligature was sufficient, in others as many as four were applied. In none have there occurred any symptoms of phlebitis; the varicose veins have been obliterated, and the ulcers speedily cicatrized; and in one of the patients whom I saw six months after the operation, there was no relapse. Still, however, I do not think that this method is likely to be so successful in all cases of varices of the lower extremities as in those of varicocele."—*Brit. and For. Med. Rev., from Bulletin Génerale de Thérapeutique.* Mars, 1840.

*New Plan for Amputating through the Tarsus.* By M. C. Sedillot.—The author proposes by this plan to remedy the inconveniences which have often followed the operation of partial amputation of the foot, as practised by Chopart and modified by Lisfranc and others, in consequence of the wound and the cicatrix succeeding to it having a semicircular form, with its longest diameter opposed to that of the os calcis and astragulus, and commonly extending round three-fourths or at least two-thirds of the circumference of the stump. This form of wound and cicatrix is constantly produced, whether the operator make both a dorsal and a plantar flap or only the latter. M. Sedillot's plan will undoubtedly remedy this defect, though more experience than has yet been obtained of its success on the living subject is necessary before it can be expected to supersede those in more general use. He describes it briefly as follows:

"The patient lying down, or being seated with the leg flexed on the thigh, I find the articulation by the position of the malleoli and the prominences of the os scaphoides and the posterior extremity of the fifth metatarsal bone, of which the distances from the line of articulation are well enough known. Then grasping the dorsal aspect of the foot with my left hand, at the level of the anterior extremities of the metatarsal bones, I place the heel on the edge of a table, so as to have a convenient and firm point of support to stretch the ligaments and separate the articular surfaces as soon as their ligamentous connexions are divided.

"With a small amputating knife I make a first transverse incision, beginning a few lines in front of the calcaneo-cuboid articulation, and terminating on the middle of the dorsal aspect of the foot on the outer side of the tendon of the tibialis anticus. From this latter point I carry a second oblique incision, from behind forwards and from without inwards, which turns round the inner edge of the foot at two fingers' breadth behind the metatarso-phalangeal articulation of the great toe, and is then continued from before backwards, from within outwards, and from above downwards across the plantar aspect of the foot, to the point at which the first incision was commenced. I take care always to divide the integuments of the sole with an oblique bevelled edge, so as to free them as much as possible from the cellular and adipose tissue which might retard their union. I now dissect back the inner flap, which is thus cut out to as far as the tubercle of the os scaphoides, which I take as a guide to open the medio-tarsal articulation, cut the interosseous ligament, slide the knife between the osseous surfaces, and complete the operation by dividing the deep muscles at the level of the incision in the sole."

In this operation the wound, of which the direction corresponds to the longest diameter of the articulation, extends only from the outer edge of the foot to the middle of its dorsal aspect, and though it occupies less than a third of the whole circumference of the foot, yet it is equal to the depth of the calcaneo-cuboid and astragalo-scaphoid articulations; for one of its extremities touches the anterior, inferior, and external edge of the os calcis, and the other the summit of the head of the astragalus. It is therefore on this incision that the cicatrix has afterwards to form; and the skin of the external and superior portion of the foot is alone opposed to the great internal flap, comprising its inferior, internal, and a part of its dorsal aspects. The cicatrix will therefore of necessity be linear and very small.

Another advantage is the smaller quantity of integuments which the medio-tarsal amputation requires. In fact about an inch of skin preserved on the inner side of the foot in front of the os scaphoides is sufficient to cover the bones, and thus the preservation of the foot is rendered possible in cases in which it would otherwise be necessary to amputate the leg. The facility of dividing the tendons of the tibialis anticus, and of the extensors of the first and second toes more anteriorly, is a favourable circumstance for the preventing the drawing backwards of the stump; and the wound having the same direction as the articular surfaces, is well supported, and the more disposed to remain united because the inner flap is formed of integuments which, though not very thick, are sufficiently vascular.—*Ibid, from Gazette Médicale.* April 18, 1840.

---

*Statistical Researches on Pneumonia.* By M. Pelletan.—The results in this memoir are drawn from 75 cases of purely inflammatory or bilious and inflammatory pneumonia, observed in M. Bouillaud's wards during the years 1834–5–6, with remarkable exactness and precision. After an orderly arrangement of his cases according to the date of their progress, the author deduces the following propositions:

1. Pneumonia of one lung is more frequent than double pneumonia, in the proportion of 7 to 2. The proportion found by M. Bouillaud was one double pneumonia in 13 cases; that established by M. Andral from 151 cases observed by himself was one in 15, and from 59 cases from various authors one in 7. M. Chomel in 27 fatal cases found nine cases of double pneumonia, that is, one in 3, and in another series of 32 cases he found seven with both lungs inflamed. The exact proportion, therefore, can be stated only after further researches, which must be conducted with careful regard of the age of the patient and the form of the disease; for it is found that double pneumonia is very much more common in young children and old persons than in adults; and it was more frequent than usual in those who were attacked during the course of influenza.

2. The right lung is more frequently affected than the left, in the proportion of 5 to 2. This result accords with the observations of Hales and of Sauvages. M. Chomel also observed the right lung alone affected 28 times, the left only 15; M. Andral found the right alone inflamed 90 times, the left 38; M. Bouillaud, the right 15, the left 8 times; M. Lombard, in 968 cases observed by himself, Andral, and Chomel, found 413 cases of pneumonia confined to the right lung, and 260 limited to the left, (195 affecting both lungs.) M. Gerhard and others have also proved that the difference of liability of the two lungs is still greater in young children.

3. The base of the lung is more disposed to inflammation than its apex, in the proportion of one and a half to one. This result also is confirmed by those of other authors, who all (except M. Chomel) have found a similar, though not exactly the same, proportion. M. Andral's results are very nearly the same as M. Pelletan's; M. Bouillaud's present a larger proportion in which the base of the lung was inflamed, and so do those of M. Valleix, which were drawn from the cases of young children.

4. Pneumonia is twice as frequent between the 17th and 37th years of age as at any other period of life. Previous authors differ widely in their expressions on this subject, and almost every period of life has been considered by some as especially liable to pneumonia; it is probable, therefore, that other conditions more powerful than the influence of age have hitherto obscured its real effects.

5. The most general direct cause of pneumonia, in at least seven-ninths of the cases, is cold; a fact fully acknowledged by all other authorities under whatever circumstances their observations were made, with the exception of M. Chomel, a large proportion of whose pneumonic patients could assign no cause for their illness.

6. In 13 cases out of 14 the pleura opposite the pneumonic portion of lung was also inflamed, so that there was pleuro-pneumonia. This result is also confirmed by the observations of M. Andral on the pneumonia of adults; but those of MM. Vernois and Valleix show that the coincidence is not so general in very young children.

7. The number of arterial pulsations in the minute affords no exact measure of the degree at which the pneumonia has arrived, or thereby of its severity. M. Chomel agrees on the whole with M. Pelletan on this opinion; but M. Andral and most others ascribe more importance to the indication of the pulse, and regard its great frequency as one of the most alarming signs.

8. The frequency of inspiration is a more serious and important sign for prognosis than the degree of acceleration of the pulse. In all cases, the number of inspirations affords an exact estimate of the degree which the pneumonia has attained.

9. Prostration of strength and delirium are more particularly connected with inflammation of the apex of the lung. Delirium is one of the most serious symptoms, and in all M. Pelletan's cases was followed by death. The first of these assertions is on the whole, though not very distinctly, confirmed by the observations of others; the last accords with the opinion of all, though it is allowed that delirium may occur in the course of pneumonia from circumstances scarcely connected with that disease, and not indicate any peculiar danger.

10. In these 75 cases there were 21 of bilious pneumonia, of which 16 occurred in the cold months, and 5 in the warmer season. It does not appear, therefore, that an elevated temperature has any influence in predisposing to the bilious form of the disease. Temperament seems more important in this respect; for in 20 of these bilious cases 11 were of a bilious or sanguineo-bilious habit. It was remarkable that in 17 of these 20 cases the pneumonia affected the base of the right lung, and in only three that of the left. Purely antiphlogistic treatment was as successful in these as in any other cases. All these cases of *bilious pneumonia* were actively inflammatory, and only differed from the rest in being accompanied by symptoms of affection of the liver, and especially by jaundice.

The second part of the memoir relates to the effects of the curative means employed. All the cases were treated on M. Bouillaud's plan of repeated bleedings; the amount of blood varying from 10 to 17 palettes (of about six ounces each,) were always drawn in the first three or four days after the patient's reception into the hospital. In pneumonia of one lung, of the first and second degrees of severity thus treated, recovery was the general rule, and death a rare exception; only two cases in 55 terminating fatally. In double pneumonia similar treatment was followed by recovery in 11 cases out of 16. In all the cases, recovery took place between the fifth and seventh days of treatment, and between the ninth and thirteenth of the duration of the disease. Pneumonia of the third degree of severity was unaffected by this method of treatment; and both the patients on whom it was employed died.

The results of M. Louis's practice, as well as that of MM. Rilliet and Barthez among children, do not appear so favourable to this plan of bleeding, *coup sur coup;* but its value is entirely confirmed by M. Husson's cases, in which of 43 pneumonic patients bled from once to eleven times each, only three died, and these had the disease under very unfavourable circumstances.

In reference to the duration of the disease also the bleeding system appears equally advantageous; in these cases it continued from nine to thirteen days, while in 50 of M. Louis's, treated with less activity, the average duration was fifteen days; and twenty days in those who were bled between the fifth and ninth day of the affection.

Of blisters, M. Pelletan says that whem employed after sufficient abstraction of blood, they never increased the fever, but appeared to have advantageous results; an observation which is on the whole confirmed by the opinions of other good authorities, from which it is deducible that counter-irritation is frequently beneficial in pneumonia affecting adults, always useful in that of old people, and sometimes so in that of children.—*Ibid., from Bulletin de l'Académie Royale de Médecine.* Janvier 31, 1840. (*Abstract of the Report by M. Rayer.*)

---

*Calculus, weighing* 23½ *ounces, formed in the Urethra.* By Dr. Da Luz.—J. L., æt. thirty, a fisherman near Lisbon, had had a difficulty of passing urine from his infancy. From the same period also he had had a hard tumour in the perineum; and at a later date a fistula, which had healed spontaneously. The tumour, however, continued to increase, though without producing any inconvenience except from the distension of the parts around it, which becoming at last excessive, the patient came to the Hospital of San Joseph, at Lisbon.

At this time (1836) the tumour which projected into the perineum had a diameter of five and a half inches; its form was nearly that of a large pear; it occupied the whole scrotum, and caused great distension of its integuments. The testicles lay one on each side of the tumour; the penis in a normal state was in its front. The perineum, which was hard and very prominent, presented several irregular cicatrices, and here and there excoriations from the contact of a sedimentous, purulent, and fœtid urine, which constantly flowed by drops from the meatus. The catheter easily detected a foreign body in the urethra.

Two deep semi-elliptical incisions being made in front of and below the tumour, the base of a calculus was exposed on which there was a furrow corresponding to the septum scroti, which gave it a bilobed form. The incision being then prolonged towards the perineum the stone was drawn out with forceps, leaving a great cavity, at the bottom of which the prostatic portion of the urethra and the neck of the bladder were seen so dilated that the operator was able to introduce his finger into the bladder. The patient left the hospital in twenty-nine days; the operation was followed by no bad consequences, but a fistula remained in the perineum which healed in the course of the following year.

The calculus, which there is every reason to believe had passed when small from the bladder into the urethra where it became fixed, was, as already said, pear-shaped; the smallest portion of it corresponded to the neck of the bladder, the larger end was enveloped by the membranes of the scrotum; two depressions were observed in its sides, where the testicles had rested; and two grooves in the under surface, one of which fitted upon the septum scroti, while the other extended along the whole length of the stone, and permitted the flowing of the urine. The composition of the calculus was phosphate of lime; it measured five inches in its longest diameter, and three and a half inches transversely; its weight was twenty-three and a half ounces.—*Ibid.*, *from Revue Médicale.* Février, 1840.

---

*Extirpation of nearly the whole of the Clavicle.* By Prof. Regnoli, of Pisa.—The subject of this operation was a carrier, thirty-four years old, who had always enjoyed good general health. In August, 1838, while lifting a sack of grain, he felt a pain in his left shoulder which ceased after a short time. Not long after, however, it reappeared in the region of the clavicle, affecting him especially every time that he returned to his work; and at the end of about ten days it became incessant and prevented his sleeping. The suffering part, as well as those adjacent to it, now rapidly swelled, and the application of leeches and other means were ineffectual in restraining the inflammation that ensued; suppuration took place, and the matter made its way out by ulceration through the skin. In November, when the patient presented himself at the Clinic at Pisa, a considerable extent of the clavicle was found to be in a state of necrosis; the patient was emaciated and had hectic fever; and after incisions over the dead bone and some other means had been employed without any benefit, it was determined to extirpate the clavicle.

For this purpose, the patient being seated, an incision was made through the track of the several ulcers over the clavicle and prolonged to each extremity of that bone, so as to pass a few lines over each, and especially over the sternal end. The bone being thus exposed a portion of its diaphysis was found almost isolated, and this being seized with strong forceps was with little difficulty extracted. The two extremities still remained; the sternal was in a state of necrosis, but the acromial appeared healthy; the former was disarticulated by cutting with scissors, and the latter (which was but a small portion) was left. The operation was rendered peculiarly difficult by the tissues having lost all their natural appearance, so that it was impossible to recognise any anatomical relations for the guidance of the knife. There was no bleeding from any considerable vessel, nor was any artery tied; the subsequent progress of the case presented nothing remarkable, except that in the inflammation which followed the operation the acromial portion of the bone became necrosed, and some pieces of it were extracted. The extirpated portion of bone was ultimately replaced by a dense tissue of fibrous consistence: the patient was enabled to move his arm in all directions without any difficulty; and, with the exception of slight weakness of the limb, the consequence of its want of exercise, every thing was restored to its normal condition.—*Ibid.*, *from Annali Medico-Chirurgici di Roma.* Vol. I., 1839.

---

*Remarkable Case of Hernia of the Fallopian Tube, with Dropsy of the Hernial Sac.* By M. A. Berard.—Madame B., æt. forty-five, has habitually enjoyed good health, complaining only within the last few years of occasional weakness in the loins, and transient pain in the lower part of the abdomen; has had two natural accouchements, and has never worked at any laborious trade. Two years since she perceived a small tumour, disappearing on pressure, in the right groin, for which she was recommended by her medical attendant to wear a bandage; this advice she neglected; the tumour slowly increased in size, continuing to be reducible. In December, 1837, its growth became more rapid, and the abdominal pain more acute than usual. At this period, M. Bérard and another practitioner ascertained the following particulars: in the right groin is seated a tumour larger than a hen's egg, stretching somewhat towards the abdomen and right labium, with a broad base and even surface, except internally and superiorly, where a mammillary body about the size of the top of the finger protrudes above the rest; here, too, the skin is adherent, exceedingly attenuated, and slightly bluish; elsewhere the integuments of the swelling preserve their natural characters. The tumour is painless, irreducible, and unchanged in size by long-continued pressure in various directions; percussion shows that it contains no gas; it is manifestly fluctuating in every part, and, judging from its perfect transparency, is filled with serosity. The patient affirms that the tumour still returns into the belly, when she has lain for some time in bed. A hard round body, of the size of a turkey's egg, protrudes above the pubis; by a vaginal examination this is found to be developed in the body of the uterus. From these facts we inferred that the patient had one or more fibrous tumours in that organ, and that on these depended the abdominal pain and weight in the loins and pelvis. We set aside all idea of strangulated hernia, and considered the inguinal tumour caused by a serous cyst developed in the part, or by an old hernial sac, closed by adhesion at the neck, and affected with dropsy. The patient becoming anxious for its removal, M. Marjolin's opinion was had; and the diagnosis now made was: femoral hernia of long standing; dropsy of the sac, which contains, besides, some abdominal viscus, probably a piece of omentum; complete adhesion of the latter with the neck of the sac, whereby the passage of the contained liquid into the abdomen is prevented. The tumour was now punctured with a trocar at its most prominent point, and from six to eight ounces of citron-coloured, frothy serosity, becoming gelatinous by heat, drawn off. After this evacuation a round body, of the size of a small nut, and irreducible, was distinctly felt in the femoral ring, and ceased to be felt behind the crural arch. Compresses steeped in aromatic wine were spread on the sac, to excite adhesion of its sides. In the afternoon a fit of shivering came on, and the tumour grew painful; the following morning the latter had refilled; skin red and hot; general prostration; twenty-five leeches to the tumour. *Third day.* Abdomen painful and distended; vomiting. The tumour, incised freely, discharges much lactescent serosity. *Fourth day.* Erysipelas about the tumour; thirty leeches. Pallor, pulse small in the day, rose in the night. *Fifth day.* The sac suppurates. *Sixth day.* Symptoms of peritonitis evident; mercurial friction, blisters to the thighs. *Seventh day.* Death. *Dissection.* Sero-purulent effusion in abdomen, false membrane on the intestines, &c. The interior of the cavity of the tumour is lined with an albuminous exudation, and communicates by a perfectly free opening with the peritonæal cavity, behind Poupart's ligament; this is evidently the neck of a hernial sac: the opening is two or three lines wide, and corresponds to the femoral ring. *The hernial sac contains nothing but the Fallopian tube in a state of considerable hypertrophy;* there is no adhesion between the tube and the interior of the sac, but the former is closely united to the anterior part of the circumference of the latter; in the space where these parts are not adherent there exists a free communication between the peritoneum and the interior of the cavity containing the serosity. The ovary of the right side occupies its usual position in the pelvis; the tissue of the uterus, otherwise healthy, is distended by an enormous fibrous tumour. From the appearance of the parts it is very possible that the liquid in the sac may have gradually oozed during the night through the opening; though the sudden pressure used in the taxis, by forcing the displaced tube into the narrow passage, may have stopped the latter up completely. In this manner the assertion of the patient respecting the disappearance of the tumour in the night, is reconcileable with the fact that it was irreducible by art.

[Cases of hernia of the Fallopian tube without the uterus or ovary are extremely rare; the erudite editor of the journal from which we make this extract has only been able to discover two such cases, and the nature of one of these cases is not wholly incontestible.]—*Ibid.*, *from L'Expérience.* Avril, 1839.

---

*Reduction of Strangulated Inguinal Hernia, by the method of Dr. Hesselbach.* By Dr. Lyncker.—[The following case is well worthy of consideration: it belongs to the rude, rough, common-sense surgery of the old time, now, perhaps, too indiscriminately banished from practice.]

Dr. Lyncker attended (April 25) a cachectic old woman, who had suffered for four days from a strangulated inguinal hernia. The belly was swollen, but not painful; the seat of the rupture was painful, but without any other perceptible change. The taxis was ineffectually employed. Leeches, cold applications, and tobacco clysters, were made use of. On the following day the taxis was again resorted to, but without effect.

During the night of the 26th, and on the 27th of April, there came on fæcal vomiting, with great restlessness, anxiety, and debility; but there was no change in the situation of the hernia. The taxis was again carefully employed, but made no impression on the swelling. The patient would not submit to an operation. Dr. Lyncker bethought himself of the method of reducing intestinal ruptures, thus described by Dr. Husselbach. "A strong man should place himself at the end of the bed on which the patient is lying, and, placing the legs of the patient over his shoulders, so that each knee of the patient shall rest upon one of his shoulders, his feet hanging downwards, shall then raise up the patient. The thighs of the patient are thus drawn upwards, his head and body resting upon the bed. In this position the taxis is to be repeated."

By placing the patient in the position above described, and re-employing the taxis, the ruptured bowel was suddenly reduced.—*Ibid.*, *from Wochenschrift für die Gesammte Heilkunde.* 1839.

---

*On Amputation of the Penis.* By M. Barthelemy.—"I published in 1829 the description of a new mode of proceeding in cases of amputation of the penis, the peculiarity of which consists in the introduction, before performing the section, of an elastic gum catheter, which is made to abut against the posterior wall of the bladder. Objections have been made to this plan: it has been said that the end of the catheter left in the bladder, after the amputation of the organ, must slip into that viscus; experience shows, on the contrary, that it springs forward from the elastic reaction of the bladder. The plan has also been declared useless; but experience destroys this objection also, for after the division of the penis, the urethra sinks inwards in such a manner that it is always difficult, and has been *sometimes impossible* to find its orifice. The following facts prove this: M. Béclard, of Strasburg, amputated a penis in presence of M. Casimir Broussais, and being utterly unable to find the orifice of the urethra, was obliged on the following day to puncture the bladder through the rectum; the canula having accidentally escaped, it became necessary to puncture above the pubis, an operation subsequently several times repeated; the bladder was in a deplorable state, when the patient fell a victim to a most seasonable attack of small-pox. M. Gimelle having amputated a penis, was unable to find the urethra, and his patient died in consequence of infiltration of urine. MM. Mirmont and Bury were, in two cases, (and I have read of a similar one in the Lancet,) unable to find the canal for a quarter of an hour. Whether this difficulty depends upon retraction of the mucous membrane of the urethra, like that observed to take place in the arteries of lacerated parts, or upon investment of the orifice of the canal by the neighbouring spongy tissues, is not easy to determine; the important point is that such difficulty arises, and that it may be obviated by the precaution I recommend. In the performance of the operation it must be borne in mind that a pliable catheter is requisite, and that this must be carefully made to abut against the posterior wall of the bladder. An assistant should be placed to the right of the patient, and placing his left hand on the pubis, press the tissues by means of the index finger and thumb with some force against the catheter, while with his right he supports the part of the instrument protruding from the urethra. If the disease have spread too close to the pubis to admit of the fingers being placed as just directed, the canal may be pressed from below upwards behind the scrotum. The operator then cuts with a small amputating knife through the penis and catheter at a single stroke. The assistant then ceases to press upon the latter, which immediately springs forwards; the operator then draws it out about three inches, and fixes it in its place in the ordinary way." Five cases are referred to wherein this plan was pursued with success.—*Ibid.*, *from Gazette Médicale de Paris.* Nov., 1839.

---

*Remarks on the Pathology and Treatment of Croup.*

To the Editor of the Medical Gazette.

*Sir*,—My object in a former communication was to show the benefits accruing from a *combination of opiates with antimonial emetics very early* in croup. My convictions are strong as to the utility of the practice. I am, of course, fully aware of the benefit which every one knows to be derived from an antimonial emetic alone; but from the good effects produced by the anodyne, I am persuaded the combination will greatly enhance the success. I purpose, with your permission, laying before the profession a few notes of cases of croup, illustrating this point, and subjoining one or two others as gleanings in the pathological anatomy of the disease under consideration.

*Case* I.—T. H., æt. eighteen months, stout and healthy, was seized with very unequivocal symptoms of croup on 16th of Feb., 1837; tonsils red and swollen, but no effusion or membrane on them; attack preceded by catarrhal symptoms of considerable severity. He was ill ten hours before I saw him; he had immediately a quarter of a grain of tart. emetic, a tea-spoonful of syrup of poppy, and a dessert-spoonful of water; this was repeated twice, at intervals of fifteen minutes, before profuse vomiting took place. During this period a leech was put upon each foot: these being active, dropped off in twenty-five minutes, and the child put into a hot-bath, in which situation he vomited most freely, became pale, drowsy, and exhausted, after being fifteen minutes in the bath: he was previously fretful, and coughing and crying in a most distressing degree; all of which subsided, and he fell into a calm deep sleep. A dose of calomel in a little jelly was given before putting him to rest; he had a long, quiet, deep sleep, with little coughing, and no crying; the calomel purged him. His voice, though hoarse, was free from croupy sound, or nearly so. Two grains of calomel, and three of Dover's powder, were given every six hours, and appeared to mitigate his cough, as also did holding his head over a deep vessel, a jug, containing hot water. His gums were scarified, and the counter-irritation of equal parts of lin. ammon. and ol. terebinth. applied on a bit of surgeon's lint to the proper part of thorax, seemed to accomplish a cure. He completely recovered, and continued stout, thriving, and healthy, till the 17th of April next year, (1838,) when he forms the subject of

*Case* II.—T. H. was now two years and a half old; though healthy, has ever since the former attack of croup had a croupy sounding cough when vexed; and on taking cold he always seemed to his parents to be threatened with croup, which they combated by their own skill.

Ten days ago he took measles with considerable cough, which three days ago became croupy. Common antimonial emetics, baths, leeches to feet, purgatives, and a blister, during these three days were tried, but in vain; he is now decidedly affected with well-marked croup; breathing quick and oppressed; pulse 140; skin hot; thirst; tongue white.

To abbreviate details, I would state that every effort was made to save the child by emetics with and without anodynes; bleeding, baths, and blistering; sulphate of copper was freely administered; mercurial inunction and mercurials by the mouth were steadily persevered in till the gums were decidedly affected.

On the 19th, the following is the notice of the physical signs of the thorax:—Inspiration more difficult and shorter than expiration; dulness on percussion on some points, but not universal; natural at upper and anterior parts; respiratory sound extinct on lower and posterior parts of left lung, though percussion is natural there.

22*d*. A whitish coating over all inside of throat and fauces, and on tongue, which is spotted-like; barking sound of cough and rapid breathing; percussion of chest natural; vesicular respiratory sound gone on right side; bronchial breathing still heard.

26*th*. Besides other remarks, crepitant râle to a considerable extent on right side, but percussion nearly natural over all chest; is sinking; several mouthfuls of a fluid-like prune-juice in colour, forcibly coughed up when I was present, devoid of all smell.

27*th*. Convulsive fits and death.

He had no delirium throughout; senses acute till night of 26th. Never any blood or films coughed up or expectorated; there was a dirty white stuff on the tongue and fauces, about consistence of paste, but granular-looking, day before death. He was not apparently distressed in his breathing at the last, nor were the lips livid to the degree of those dying of croup; he mostly swallowed, instead of spitting out what came up from his air-passages.

*Sectio cadaveris.*—11 A. M. 29th April: little change in expression of countenance, which was placid; a considerable quantity of fat under the skin every where, though not much in the internal organs; muscles of a colour approaching to slight purple; a considerable quantity of fluid blood in large veins—the jugular, the subclavian, and the thoracic branches thereof. The lungs filled the chest, were of a mottled light purple and buff colour at upper parts; the summit of upper lobe on right side had many bullæ of emphysema in it; there were many fine firm membranes, of a filamentous appearance when stretched, obviously of long-standing, binding the lung to the pleura costalis, particularly the upper lobe; the lower lobe being glued to the pleura costalis by lymph, yellow, soft, and very easily broken, doubtless quite recent. The parts of the pleura costalis not adhering had many points of redness and vascularity, and there were six or seven drachms of serous fluid in it; the lower and posterior part of the right lung's surface was mottled, or spotted deep red; felt firm and liver-like, heavy, and its sharp margin covered by a recent layer of lymph; the upper lobe was distinctly emphysematous, and there were several irregular small pouches or vesicles of air, the walls of which consisted of the pleura pulmonalis, the air-cells having given way. The parenchyma of this portion was of a lake red, mingled with a reddish buff; crepitated when cut into, and swam buoyantly in water; the lower lobe was consolidated at many points of considerable size; sank in water, and was infiltrated with yellow, diffluent, puriform matter, easily torn and broken down; its section presented a somewhat mottled grayish appearance; it did not pit on pressure. The air tubes or bronchia still potent and obvious in the midst of it. *The left cavity of thorax:* pretty extensive adhesions of the pleura pulmonalis to the pleura costalis, by friable yellow lymph and a little serous fluid: an ounce and a half in cavity. The summit in left lung was crepitant, swam in water, and was tolerably free from disease, but the lower lobe was infiltrated with puriform matter, consolidated, and sank in water heavily; lost the crepitation on handling or cutting, and its substance was of a dark venous-red appearance. In both lungs there were portions in very various stages of phlogosis.

The coating observed previous to death on the tongue and fauces had disappeared, nor was any visible on the gullet, epiglottis, and the margins of the glottis. The epiglottis was thicker than natural, and the margins of glottis were red as red currant jelly, swollen to the thickness of half a common lead pencil all round the margin of the chink, forming, as it were, a collar or rounded edge, instead of the sharp margin of these parts in their natural state. The surface of this swollen margin was abraded at some small points, and on its surface were several small vesicles or blisters. It felt soft and fleshy; no fluid flowed on cutting into it. The lining membrane of the larynx was of a red curant-jelly-like colour, several vessels seen on it, and some dirty mucus with specks of the lining membrane intermixed, several patches of which, very thin and very pale, and not larger than herring scales, were still firmly adherent to the mucous lining of the air-tubes. The redness and the small patches extended down the trachea and bronchi. None of the prune-juice looking stuff, which he coughed up before death, could be seen in the air-passages.

The heart was healthy; right cavities contained primrose yellow clots, about the full of an ounce measure, and much purple serum flowed away on cutting off the apex of the heart; they had long tails extending into the venæ cavæ and into the pulmonary artery, which, when drawn out, might by fancy be likened to worms. There was a little of the same coagula in the left side, but not one-third so much as in the right.

Serous membrane of *abdomen* quite natural throughout; liver paler than usual. Gall-bladder very full, and of a dark green colour.

Mucous lining of alimentary canal had every evidence of a phlogosed condition in many parts. The stomach had much dark-greenish fluid in it; spleen and pancreas sound; kidney, ureters, and bladder, healthy. We were not permitted to open the head.

*Case* III.—R. B., æt. two years, a healthy child, but had a smart attack of diarrhœa for some days, which has left him weakened and languid. About 11 P. M. of 3d of August he was observed suddenly to cough and breathe with a croupy sound, which the whole family knew too well from his sister's late fatal attack. *He has had no cough or other catarrhal symptoms*, and the first sound of a croupy kind was heard in his waking out of sleep and suddenly coughing and breathing with the brazen clanging sound.

I saw him at half past 11 P. M., when he had no other symptom than the barking, croupy cough, and when peevish back-draughts of a hoarse, croupy character. Every function in a healthy state; no fever; cheerful and lively, and played in good spirits when in the hot bath, into which he was instantly put. He had a dessert-spoonful of a strong solution of tartar emetic, and eight drops of the sol. mur. morph. in it. The emetic solution was repeated in fifteen minutes with four drops of the anodyne. He was kept forty minutes in the hot-bath, and vomited freely several times. The croupy sound abated; he soon fell into a calm slumber.

11 A. M. of 4th August.—He had a very quiet night, and had only one or two croupy coughs. Had two doses of Dover's powder, two and a half grains each. One or two patches of dirty lymph on posterior fauces, and a large one on left tonsil, which is red and swollen. A dose of castor oil has purged freely. No constitutional symptoms.

1 P. M. Croupy symptoms have returned as well marked as ever; and at 1 P. M., after a consultation with Dr. James Watson, he had an emetic dose of the antimonial solution every hour: in the first dose I gave him six drops of the solution of mur. morph. Two grains of calomel every two hours were also given. He was kept in bed, and a counter-irritant embrocation, (ol. tereb. et lin. ammon.) applied to chest.

*Vespere.*—He has vomited freely; all patches off the throat; has slept a great deal all day, and free from cough and croupy symptoms. Dr. Watson, whose kind and efficient attentions he now had, (for I was at St. Andrew's,) continued the calomel; and on Wednesday evening I found him free from complaint, though very weak.

*Remarks.*—This case may be viewed by some as differing from the type of real croup; it might be said that it came on in too sudden a manner, and wanted the precursory symptoms of catarrh or bronchitis, which usher in certainly a vast proportion of cases of cynanche trachealis. I should have thought so too, unless I had evidence from experience of the accession of real croup being as sudden and unexpected as in the case just related.

*Case* IV.—A girl, four years of age, full and healthy, was somewhat exposed to a cold east wind; she was put to bed at 8 P. M., in high health and spirits, and was observed to be free from complaint as late as 11 P. M. that evening. About 3 A. M. of following morning she awoke suddenly, with sense of suffocation, intense pain in ear, croupy breathing, and other evident marks of great distress. I saw her about 4 A. M. of 4th March, with every symptom of croupy breathing and cough; much earach; tonsils reddish and slightly swollen, but presented no other morbid appearance. She had an emetic of sulphate of copper, and the same medicine continued in smaller doses; leeches applied to throat, and warm bath; calomel was given in the forenoon, and repeated during the day; with the calomel in the evening a dose of Dover's powder was given. Considerable relief ensued; the medicines were continued, and a blister was applied to top of sternum. On the 5th I was called out of town, and requested the parents, in case she was no better, to ask the advice of one of my professional friends. On the 7th I found her, after the steady use of the sulph. cupri, the calomel and Dover's powder, considerably freer from croup; cough and breathing less strepitous. On the 8th symptoms were aggravated, and a thick layer of dirty white lymph on whole of tonsils, uvula, and pillars of fauces, and extending down as far as the eye could reach; breathing croupy, loud, and laborious. A fearful attack of suffocation came on, followed by convulsions and coma, in which state she continued till evening of same day, and died. Leave could not be obtained to open the body.

I could adduce other cases in which croup of an inflammatory nature came on with equal suddenness, so that I cannot admit its sudden advent to be a sufficient mark that the case is not cynanche trachealis, or of an inflammatory character, but one of a spasmodic nature. Nor can I avoid relating the following case, to show that *in some cases of inflammatory croup febrile symptoms are very late in making their appearance*, and that their absence should not throw us off our guard, so as to lead us to mistake the disease because there are no febrile symptoms present, an error into which the description of some authors might lead the unwary.

*Case* V.—W. R., a boy six years of age, full habit, though labouring under hooping cough for twelve weeks, and sent here from Aberdeen for change of air.

30*th October*, 1834.—Three days ago his cough put on the croupy sound, and his breathing became *wheezing*, particularly at night; but he continued playful and took his food, though rather restless and disturbed by cough last night. This morning he took his porridge at breakfast, and came, to all appearance, free from complaint, into the room to have my advice. This is my note of the case:—"Distinct croupy breathing and croupy cough; appetite good; pulse natural; bowels moved by castor oil; tongue slightly white; skin cold and moist." Notwithstanding the entire absence of constitutional or febrile symptoms, the decided character of the croupy sound induced me to point out to the friends of the boy my alarm and his risk, and to ask a consultation. Dr. James Watson saw this patient along with me; our chief reliance was placed on antimony and mercurials; leeches and a blister were also had recourse to.

On 31st, (the fifth day of the disease,) there is the following note:—"A good night after free vomiting; but at 7 A. M. began to breathe with more difficulty, and I now (11 A. M.) find it very much more oppressed and more wheezing; pulse ninety, soft; skin natural. No visible affection in fauces; he points to larynx, and says it is painful; sounds of respiration and on percussion quite natural."

*Vespere.*—After six leeches to the throat, which bled well, I find his breathing more loud, twenty-six times in a minute; inspiration and expiration equal in length; he is restless, but occasionally sleeps a little; considerable visible movement or heaving of chest; sound of chest on percussion quite natural; respiratory sound extinct in some parts, but wherever heard it is natural. Now and then there is a fit of coughing, which is like a bark, and very brazen and clanging, and has a back draught.

*Nov. 1st.*—Blister rose in five and a half hours; a very restless night, during which breathing difficult, and he "vomited" or coughed up a film, which, on inspection, had the consistence of lymph, one broad piece of a quarter of an inch broad, nearly one inch in length, and having several tails or branches attached to it, obviously from the air-tubes, though they were not entire or tubular themselves. About 9 A. M. to-day his feet became cold, his breathing slower and slower, countenance livid and cadaverous, and he sunk without a convulsion or struggle at half past 10 A. M. He was quite acute and sensible till within two hours of his death. He felt relieved after vomiting the film.

On inspecting the trachea we found it inflamed, and in part covered with a layer of lymph of various breadths, and its continuity broken at two or more points, there was no lymph in left bronchus or in larger branches near it, probably the site from whence the film coughed up had been separated. The film or layer of lymph penetrated, as far as we could trace it, into the tubes in the parenchyma. There was a considerable quantity of fluid (serum) in the substance of the lung, but no inflammation in it, or in pleura; some portions of the lung emphysematous.

I cannot doubt, after having seen such cases as these recited above, that croup or tracheitis may come on to all appearance in a most sudden and unexpected manner, and, until a very advanced period of its progress, may be unattended with febrile symptoms. I would most respectfully suggest, that the following sentence from the writings of an eminent author should have been qualified, lest (as I fear it will) so unguarded a statement lead into error. "From spasm of the glottis, or purely spasmodic croup and hysterical affections simulating it, inflammatory croup may be distinguished by the presence of febrile symptoms, the less sudden and more permanent character of its attack, and other points of its history." Against a statement so likely to mislead the unwary I beg to raise my testimony. With your permission I shall transmit one case more of this fatal disease, with a few remarks.—I am, sir,

Your obedient servant,

ALEX. J. HANNAY, M. D.

---

*On the Microscopic Characters of the Menstrual Blood.* By Dr. BUROW, of Konigsberg.—Dr. Burow examined twelve ounces of menstrual blood which had been retained in the uterus by an imperforate hymen. The blood was of a dirty reddish-brown colour, of the consistence of syrup, very adhesive, and perfectly destitute of smell. It abounded in albumen, and was very little susceptible of putrefaction.

When looked at under the microscope almost all the blood-globules were seen to have lost their regular form, and to resemble those granules which may be observed in pus which has been for a long time exposed to the air, or retained within the cavity of an abscess. These blood-globules were suspended in a transparent fluid. On stirring the blood for a considerable time no change perceptible to the eye was produced, but under the microscope a great number of delicate transparent lamellæ were seen floating in the serum, and these Dr. Burow regards as portions of fibrine, which substance is sparingly present in menstrual blood.—*British and Foreign Medical Review*, *from Muller's Archiv.* 1840.

